# Late-Onset Episodic Weakness With Hyperkalemia Suggestive of Hyperkalemic Periodic Paralysis

**DOI:** 10.7759/cureus.114543

**Published:** 2026-08-14

**Authors:** Hemant K Pandey, Kumaraswamy Sivakumar, Kinsey J Gudenkauf, Rishil Kadakia

**Affiliations:** 1 Neurology, Brain and Spine Center, Chandler, USA; 2 Neurology, Barrow Neurological Institute, Phoenix, USA; 3 Neurology, AT Still University KCOM (Kirksville College of Osteopathic Medicine), Mesa, USA; 4 Neurology, University of Illinois Urbana-Champaign, Urbana, USA

**Keywords:** channelopathy, hyperkalemia management, hyperkalemic periodic paralysis, ion channelopathy, periodic paralysis

## Abstract

A rare late-onset presentation of episodic weakness associated with hyperkalemia is described in a 72-year-old male with concurrent early-stage chronic lymphocytic leukemia. He experienced frequent episodes of generalized muscle weakness accompanied by loss of speech, shortness of breath, and fatigue, with a documented serum potassium level of 6.5 mmol/L obtained within 30 minutes of an attack. Treatment with acetazolamide and dietary modification was associated with a substantial reduction in episode frequency and improved functional status, although persistent fatigue remained. The clinical presentation was suggestive of hyperkalemic periodic paralysis, but a definitive diagnosis was limited by the patient's exceptionally late age at onset, negative genetic testing, lack of specialized exercise testing, and absence of electrophysiologic testing during an attack. This case emphasizes the importance of considering hyperkalemic periodic paralysis in the differential diagnosis of episodic weakness while simultaneously evaluating alternative neuromuscular, metabolic, hematologic, and paraneoplastic causes. Treatment response to acetazolamide and dietary modification was considered supportive of but not necessarily diagnostic of an inherited channelopathy.

## Introduction

Hyperkalemic periodic paralysis is a rare autosomal dominant skeletal muscle channelopathy most commonly associated with pathogenic variants in the SCN4A gene. These mutations alter the function of the Nav1.4 sodium channel, prolonging channel opening and disrupting sodium and potassium ion transport, which impairs muscle membrane excitability and results in episodic muscle weakness or paralysis, often accompanied by hyperkalemia [1]. The disorder has an estimated prevalence of approximately 1 in 200,000 individuals and most commonly presents during the first or second decade of life [1]. Adult- or late-onset presentations are exceptionally uncommon and may be overlooked because of their atypical timing. This case describes a 72-year-old man with chronic lymphocytic leukemia (CLL) who presented with recurrent episodic weakness followed by an episode of hyperkalemia, a presentation suggestive of hyperkalemic periodic paralysis but without genetic or electrophysiologic confirmation.

Episodes of hyperkalemic periodic paralysis are classically precipitated by rest after strenuous exercise, fasting, emotional stress, cold exposure, potassium-rich foods, or prolonged immobility. Weakness typically lasts 15 minutes to 2 hours with complete recovery between attacks, although fixed proximal weakness may develop over time. Diagnosis requires integration of the clinical phenotype with supportive laboratory and electrophysiologic findings and, when available, molecular confirmation. However, approximately one-third of clinically suspected patients do not have an identifiable pathogenic SCN4A variant, making the diagnosis not reliant on genetic testing alone [2]. In this patient, the diagnosis remained uncertain because genetic testing was negative and specialized exercise testing or electrophysiologic testing during an attack was not performed. The presence of CLL substantially broadened the differential diagnosis. CLL may contribute to hyperkalemia through pseudohyperkalemia associated with marked leukocytosis or true hyperkalemia during tumor lysis, and it can also be associated with autoimmune or paraneoplastic neurologic syndromes that cause episodic weakness. Distinguishing these secondary causes from a primary skeletal muscle channelopathy was therefore a major diagnostic challenge in this case.

## Case presentation

A 72-year-old male with a history of paroxysmal atrial fibrillation presented with a 10-month history of recurrent, transient episodes of generalized muscle weakness and shortness of breath. His medical history was otherwise negative for prior neuromuscular disease, thyroid disease, renal disease, adrenal insufficiency, or episodes of paralysis earlier in life. Medication review revealed no recent changes or agents known to commonly precipitate hyperkalemia. Family history was negative for periodic paralysis, unexplained episodic weakness, or hereditary neuromuscular disorders.

An initial episode began with him having difficulty rising from a chair. During subsequent attacks, he developed profound generalized weakness, rendering him unable to speak or move his extremities while remaining fully alert. Episodes were consistently characterized by preserved consciousness without diplopia, ptosis, sensory loss, bowel or bladder dysfunction, or loss of awareness. Symptoms resolved spontaneously without residual focal neurologic deficits, although generalized fatigue often persisted for several hours. During the one observed episode in the office, the patient developed generalized weakness with hypophonia. He remained able to maintain upright truncal and neck posture, and spontaneous movement of both axial and appendicular muscles against gravity was preserved. Formal strength testing demonstrated reduced force generation, most pronounced in the hands, with markedly impaired grip strength; however, antigravity strength was maintained. Extraocular movements were intact without apparent ophthalmoplegia. Reflexes, sensation, and coordination were not assessed during the episode, and gait was not assessed because of his inability to ambulate. Between attacks, the patient was fully alert and oriented, with no evidence of large-fiber neuropathy or persistent proximal or bulbar weakness. Cranial nerve examination was unremarkable, including normal extraocular movements, and gait was stable without fluctuation or abnormalities. Strength was preserved, reflexes were normal and symmetric, and sensory and coordination examinations were intact.

Over time, the patient's attacks increased in both frequency and severity, occurring approximately every 3 days and lasting 1.5 to 2 hours. Most episodes occurred from morning to early afternoon and were frequently preceded by panting and diffuse weakness. He denied pain, sensory symptoms, autonomic complaints, or constitutional symptoms but reported anxiety during attacks because of dyspnea. Over the preceding months, he also noted mild cognitive changes, including difficulty with complex tasks and intermittent word-finding difficulty. At follow-up two months later, the episodes had become more severe and occasionally resulted in falls. He identified emotional stress as a potential trigger and reported worsening fatigue between attacks that limited previously routine activities such as exercise and golfing. Laboratory evaluation at this time revealed leukocytosis, leading to the diagnosis of early-stage chronic lymphocytic leukemia (CLL). Hematologic evaluation demonstrated no evidence of tumor lysis syndrome, rapidly progressive disease, or other metabolic abnormalities sufficient to explain the recurrent stereotyped episodes of weakness.

Nerve conduction studies of the upper extremities demonstrated prolonged bilateral distal peak latencies and reduced conduction velocities of the bilateral median and ulnar sensory nerves, along with reduced conduction velocity of the right radial sensory nerve (Table 1). Bilateral median motor nerves also demonstrated prolonged distal onset latencies (Table 2). Electromyography performed between attacks showed no active denervation, myopathic changes, or electrical myotonia (Table 3). Importantly, there was no long-exercise test, short-exercise test, provocative exercise protocol, or testing during an actual weakness episode that was documented. Therefore, the study was not designed to adequately evaluate for periodic paralysis channelopathy, and its normal needle EMG findings cannot be considered evidence towards or against hyperkalemic periodic paralysis.

**Table 1:** Nerve conduction studies of upper extremities: anti-sensory summary

Stimulation Site	Not Recorded	Peak (ms)	Normal Peak (ms)	Peak-to-Peak Amplitude (μV)	Normal Peak-to-Peak Amplitude (μV)	Site 1	Site 2	Delta-0 (ms)	Distance (cm)	Velocity (m/s)	Normal Velocity (m/s)
Left Median Anti Sensory (2nd Digit) 31.8°C											
Attempt 1	N/A	4.6	<3.6	25.9	>10	Wrist	2nd Digit	3.3	13	39.4	>49
Attempt 2	N/A	4.6	<3.6	23.1	>10	Wrist	2nd Digit	3.3	13	39.4	>49
Attempt 3	N/A	4.4	<3.6	19.9	>10	Wrist	2nd Digit	3.6	13	36.1	>49
Right Median Anti Sensory (2nd Digit) 32°C											
Attempt 1	N/A	4.9	<3.6	26.9	>10	Wrist	2nd Digit	3.5	13	37.1	>49
Attempt 2	N/A	4.7	<3.6	28.1	>10	Wrist	2nd Digit	3.5	13	37.1	>49
Attempt 3	N/A	4.8	<3.6	29.8	>10	Wrist	2nd Digit	3.7	13	35.1	>49
Left Radial Anti Sensory (Base 1st Digit) 32°C											
Attempt 1	N/A	2.8	<3	15.1	>15	Wrist	Base 1st Digit	2.0	10	50	>49
Attempt 2	N/A	2.8	<3	16.4	>15	Wrist	Base 1st Digit	0.0	0	N/A	>49
Attempt 3	N/A	2.8	<3	15.6	>15	Wrist	Base 1st Digit	0.1	0	N/A	>49
Right Radial Anti Sensory (Base 1st Digit) 32°C											
Attempt 1	N/A	2.8	<3	17.2	>15	Wrist	Base 1st Digit	2.1	10	47.6	>49
Attempt 2	N/A	2.8	<3	16.9	>15	Wrist	Base 1st Digit	0.1	0	N/A	>49
Attempt 3	N/A	2.9	<3	16.8	>15	Wrist	Base 1st Digit	0.1	0	N/A	>49
Left Ulnar Anti Sensory (5th Digit) 31.9°C											
Attempt 1	N/A	4.1	<3.2	7.5	>15	Wrist	5th Digit	2.9	11	37.9	>49
Attempt 2	N/A	4.0	<3.2	7.0	>15	Wrist	5th Digit	3.0	11	36.7	>49
Attempt 3	N/A	4.0	<3.2	7.8	>15	Wrist	5th Digit	3.0	11	36.7	>49
Right Ulnar Anti Sensory (5th Digit) 32°C											
Attempt 1	N/A	3.7	<3.2	14.8	>15	Wrist	5th Digit	2.7	11	40.7	>49
Attempt 2	N/A	3.8	<3.2	12.9	>15	Wrist	5th Digit	2.7	11	40.7	>49

**Table 2:** Nerve conduction studies of upper extremities: motor summary

Stimulation Site	Not Recorded	Onset (ms)	Normal Onset (ms)	Amplitude Origin to Proximal Site (mV)	Normal Amplitude Origin to Proximal Site (mV)	Site 1	Site 2	Delta-0 (ms)	Distance (cm)	Velocity (m/s)	Normal Velocity (m/s)
Left Median Motor (Abductor Pollicis Brevis) 30.5°C											
Wrist	N/A	4.7	<4.3	5.1	>5.0	Elbow	Wrist	4.9	24	49	>49
Elbow	N/A	9.6	N/A	4.3	N/A	N/A	N/A	N/A	N/A	N/A	N/A
Right Median Motor (Abductor Pollicis Brevis) 32°C											
Wrist	N/A	5.2	<4.3	5.4	>5.0	Elbow	Wrist	4.6	24	52.2	>49
Elbow	N/A	9.8	N/A	5.0	N/A	N/A	N/A	N/A	N/A	N/A	N/A
Left Ulnar Motor (Abductor Digiti Minimi) 31.4°C											
Wrist	N/A	3.2	<4.2	7.8	>5.9	B Elbow	Wrist	4.9	24	49	>49
B Elbow	N/A	8.1	N/A	7.1	N/A	A Elbow	B Elbow	1.4	7	50	>49
A Elbow	N/A	9.5	N/A	6.9	N/A	N/A	N/A	N/A	N/A	N/A	N/A
Right Ulnar Motor (Abductor Digiti Minimi) 31.9°C											
Wrist	N/A	3.2	<4.2	5.5	>5	B Elbow	Wrist	4.3	24	55.8	>49
B Elbow	N/A	7.5	N/A	4.9	N/A	A Elbow	B Elbow	2.0	10	50	>49
A Elbow	N/A	9.5	N/A	4.7	N/A	N/A	N/A	N/A	N/A	N/A	N/A

**Table 3:** Electromyography of upper extremities

Side	Muscle	Nerve	Root	Ins Act	Fibs	Psw	Crd	Myot	Myok	Fasic	Amp	Dur	Poly	Recrt	Int Pat
Right	Deltoid	Axillary	C5-6	Normal	Normal	Normal	Normal	Normal	Normal	Normal	Normal	Normal	0	Normal	Normal
Right	Biceps	Musculocutaneous	C5-6	Normal	Normal	Normal	Normal	Normal	Normal	Normal	Normal	Normal	0	Normal	Normal
Right	Triceps	Radial	C6-7-8	Normal	Normal	Normal	Normal	Normal	Normal	Normal	Normal	Normal	0	Normal	Normal
Right	1st Dorsal Interosseous	Ulnar	C8-T1	Normal	Normal	Normal	Normal	Normal	Normal	Normal	Normal	Normal	0	Normal	Normal
Right	Abductor Pollicis Brevis	Median	C8-T1	Normal	Normal	Normal	Normal	Normal	Normal	Normal	Normal	Normal	0	Normal	Normal
Left	Deltoid	Axillary	C5-6	Normal	Normal	Normal	Normal	Normal	Normal	Normal	Normal	Normal	0	Normal	Normal
Left	Triceps	Radial	C6-7-8	Normal	Normal	Normal	Normal	Normal	Normal	Normal	Normal	Normal	0	Normal	Normal
Left	Biceps	Musculocutaneous	C5-6	Normal	Normal	Normal	Normal	Normal	Normal	Normal	Normal	Normal	0	Normal	Normal
Left	1st Dorsal Interosseous	Ulnar	C8-T1	Normal	Normal	Normal	Normal	Normal	Normal	Normal	Normal	Normal	0	Normal	Normal
Left	Abductor Pollicis Brevis	Median	C8-T1	Normal	Normal	Normal	Normal	Normal	Normal	Normal	Normal	Normal	0	Normal	Normal

**Figure 1:**
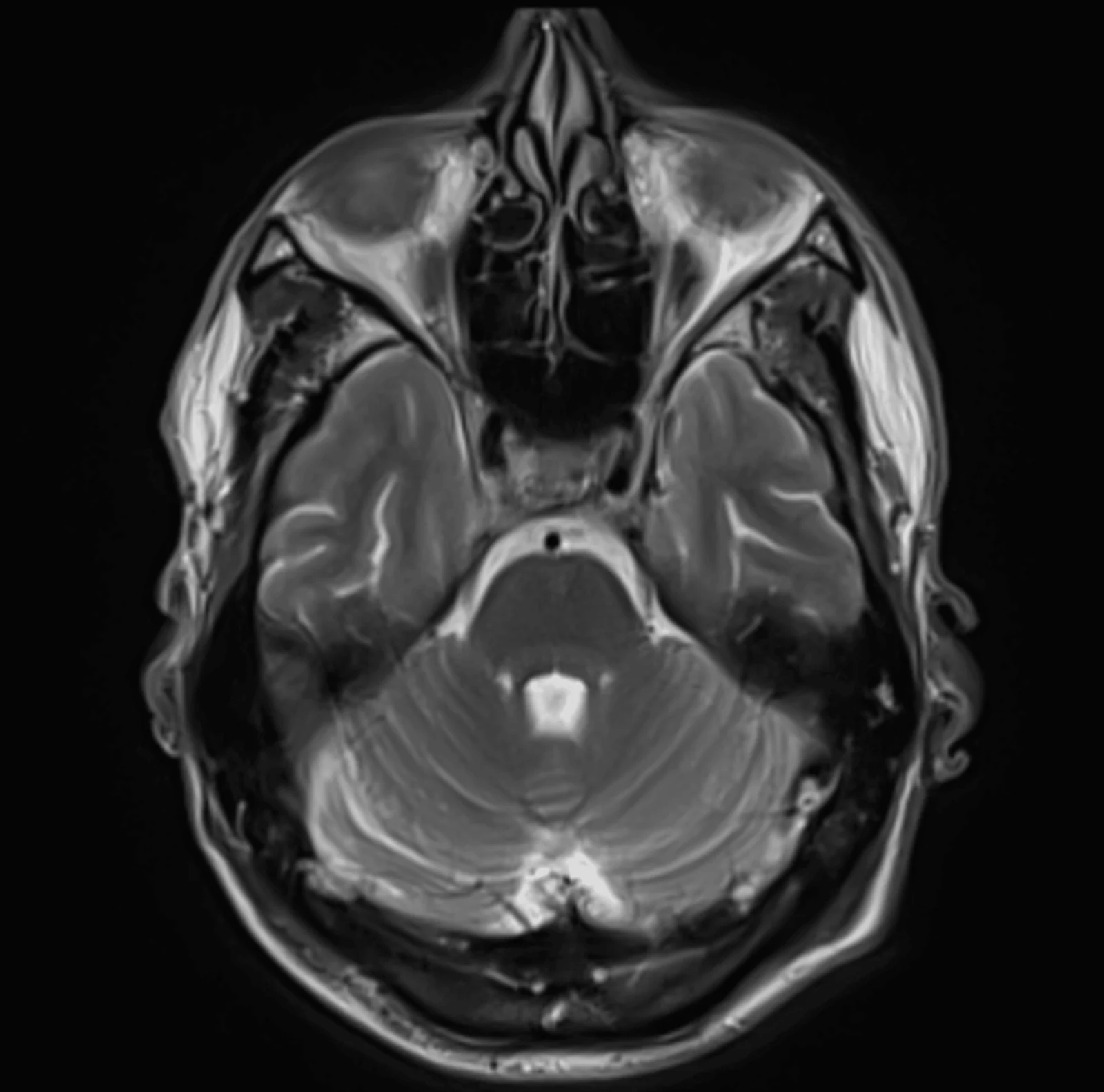
Axial T2 MRI of brain Negative for any space-occupying lesions, focal parenchymal abnormalities, or other structural CNS abnormalities. The MRI was obtained as part of the broader neurologic evaluation rather than specifically for evaluation of the patient's episodic weakness.

Thyroid function, creatine kinase, cortisol, bicarbonate, and renal function were within normal limits, while the paraneoplastic antibody panel and acetylcholine receptor and MuSK antibodies were negative. Brain MRI revealed no space-occupying lesions, focal parenchymal abnormalities, or other structural CNS abnormalities to suggest an alternative underlying pathology (Figure 1). Genetic testing for SCN4A and other channelopathy-associated genes, including CACNA1S and KCNJ2, was negative, although an incidental SMN1 carrier state was identified.

A documented serum potassium level of 6.5 mmol/L immediately following an episode was suggestive of hyperkalemic periodic paralysis, including the recurrent stereotyped attacks of generalized weakness lasting less than 2 hours, the absence of persistent objective weakness between attacks, and the substantial clinical improvement following treatment with acetazolamide, a carbonic anhydrase inhibitor [1]. The relatively short duration and high frequency of episodes were also supportive of hyperkalemic periodic paralysis [3]. However, several atypical features complicated the diagnosis. Electrical myotonia, present in approximately 50-75% of patients with hyperkalemic periodic paralysis, was not demonstrated on electromyography [4]. In addition, genetic testing for SCN4A, CACNA1S, and KCNJ2 was negative. The patient had no family history of periodic paralysis, symptom onset occurred in the seventh decade of life, and concurrent CLL broadened the differential. Accordingly, the diagnosis remained one of clinical probability rather than definitive genetic confirmation, and this case is best interpreted as clinically compatible with hyperkalemic periodic paralysis despite its atypical features rather than a genetically confirmed primary channelopathy.

The differential diagnoses included myasthenia gravis, Lambert-Eaton myasthenic syndrome, paraneoplastic neurologic syndromes associated with CLL, myopathic disorders, thyrotoxic periodic paralysis, Andersen-Tawil syndrome, and secondary causes of hyperkalemia. Although acetylcholine receptor and MuSK antibodies were negative, these findings alone do not exclude a neuromuscular junction disorder, and no repetitive nerve stimulation or single-fiber electromyography during symptoms was identified. Nevertheless, myasthenia gravis was considered less likely given the absence of persistent or fluctuating ocular or bulbar weakness, lack of ptosis or diplopia, preserved extraocular movements, preserved antigravity strength between attacks, and the complete resolution of weakness between episodes. Thyrotoxic periodic paralysis was considered given the patient's palpitations and episodic weakness [5] but was excluded by normal thyroid studies and the absence of tremor or weight loss. Andersen-Tawil syndrome was considered because of its association with periodic paralysis and hyperkalemic episodes but was less likely given the absence of characteristic dysmorphic features, cardiac arrhythmias, or pathognomonic electrocardiographic findings; KCNJ2 testing was also negative, while approximately 60% of affected individuals harbor a KCNJ2 mutation [6]. Secondary endocrine and renal causes were considered unlikely based on normal cortisol and bicarbonate levels, stable renal function, absence of hypotension or salt-induced attacks, and no relevant medication changes [2]. Only one elevated potassium measurement was documented, with a level of 6.5 mmol/L obtained within approximately 30 minutes of attack onset. Because the sample was collected at home, no contemporaneous electrocardiogram was obtained, and no serial potassium measurements were performed to determine whether the elevation preceded, coincided with, or followed the onset of weakness. Thus, this isolated measurement demonstrates an association between hyperkalemia and the episode but does not by itself establish causality. This limitation is particularly relevant in the setting of CLL and leukocytosis, which can cause pseudohyperkalemia due to leukocyte fragility. Although the remainder of the chemistry profile was similar to baseline and there was no evidence of tumor lysis syndrome, pseudohyperkalemia was not definitively excluded because paired serum, plasma, or whole-blood potassium measurements were not obtained and specimen-handling conditions were undocumented. Future episodes should ideally include promptly obtained, appropriately handled potassium measurements with consideration of paired serum, plasma, or whole-blood testing when leukocytosis is substantial.

Acetazolamide was selected for treatment because carbonic anhydrase inhibitors are considered first-line prophylactic therapy for many patients with hyperkalemic periodic paralysis [6]. Proposed mechanisms include mild intracellular acidosis, stabilization of skeletal muscle membrane excitability, and promotion of renal potassium excretion. The patient was initially started on 500 mg twice daily, which was associated with a reduction in the frequency and severity of attacks but resulted in adverse effects including worsening muscle weakness, ataxia, and severe imbalance. To improve tolerability while maintaining therapeutic benefit, the dose was tapered to 250 mg each morning and 500 mg each evening for two weeks, followed by 250 mg twice daily, which he has continued. He was also counseled to reduce dietary potassium intake to 500 mg/day, avoid sugar, limit carbohydrate intake, and gradually increase physical activity as tolerated [6]. Following initiation of acetazolamide and dietary modification, his attacks decreased from approximately every 3 days and lasting 1.5-2 hours to only 2-3 episodes over several months, each lasting approximately 20-25 minutes. He also experienced a 55-day attack-free interval and reported improved endurance and strength, fewer falls, and no longer requires rest after physical activity. Despite this improvement, he continued to experience daily fatigue, mild muscle weakness, and impaired balance with frequent stumbling, although he remains active with light exercise as tolerated. This clinical response is supportive of a possible hyperkalemic periodic paralysis phenotype but should not be interpreted as diagnostic confirmation, as symptom frequency may fluctuate spontaneously and responses to acetazolamide vary across periodic paralysis genotypes and other causes of episodic weakness. Although only 21% of patients with hyperkalemic periodic paralysis report a reduction in attack frequency [7], the patient's substantial improvement following treatment is notable but remains insufficient to establish an inherited skeletal-muscle channelopathy.

Long-term complications reported in patients with hyperkalemic periodic paralysis include persistent muscle stiffness, weakness, pain, fatigue, and an increased risk of falls [8]. Patients may also experience anesthesia-related complications because commonly used depolarizing agents can exacerbate myotonia and impair respiratory muscle function [7]. Although the diagnosis remains unconfirmed in this patient, these potential complications should be considered in future clinical and perioperative management. Ongoing care should include symptomatic management, periodic electrolyte monitoring, assessment for acetazolamide-related adverse effects, surveillance for fixed or progressive weakness, and continued hematologic follow-up for CLL. Given the absence of genetic or electrophysiologic confirmation, recurrence or evolution of his episodes should prompt further diagnostic evaluation, including potassium measurement during symptoms and, when feasible, specialized exercise or electrophysiologic testing to further assess for a periodic paralysis channelopathy.

## Discussion

This case describes a rare late-onset presentation of suspected hyperkalemic periodic paralysis in a 72-year-old man with comorbid chronic lymphocytic leukemia (CLL). The clinical phenotype was suggestive but not definitive, with numerous stereotyped attacks of generalized weakness lasting 2 hours or less, frequent association with emotional stress, preserved consciousness, and one documented serum potassium level of 6.5 mmol/L obtained within approximately 30 minutes of an attack. Current diagnostic criteria includes ≥2 attacks of weakness with a serum potassium concentration >4.5 mEq/L, or one attack in a relative with documented hyperkalemia, together with supportive features such as attack duration of less than 2 hours, identifiable triggers, myotonia, family history, and positive genetic testing when present [7]. The patient's clinical features therefore provided supportive evidence; however, several atypical features and diagnostic limitations reduced certainty. Approximately 50% of affected individuals develop weakness or paralysis before 10 years of age, with the greatest accumulation of diagnosed cases occurring by the fifth decade, although symptoms can rarely first appear later [7]. The patient's onset in the seventh decade, absence of childhood or adolescent episodes, and negative family history are therefore atypical for hyperkalemic periodic paralysis.

Hyperkalemic periodic paralysis is a hereditary skeletal muscle channelopathy most commonly caused by pathogenic variants in SCN4A, which encodes the voltage-gated sodium channel Nav1.4. These mutations impair sodium channel inactivation, resulting in persistent sodium influx, sustained sarcolemmal depolarization, loss of membrane excitability, and transient paralysis [9]. Genetic testing for SCN4A, CACNA1S, and KCNJ2 was negative in this patient, providing no molecular confirmation; however, only approximately 66% of individuals with hyperkalemic periodic paralysis have an identifiable SCN4A mutation [6], and negative testing does not exclude the diagnosis. Proposed explanations for genetically unresolved cases include incomplete detection of pathogenic SCN4A variants, disease-modifying variants in interacting genes, mosaic SCN4A mutations, and environmental or metabolic triggers such as excessive potassium intake, medications, or metabolic stressors [6]. Given the patient's unusually late onset and absence of family history, the negative genetic evaluation is particularly important, and his condition is best characterized as suspected or possible hyperkalemic periodic paralysis rather than genetically confirmed disease.

The single documented potassium elevation represents another important limitation. Although the potassium level of 6.5 mmol/L obtained within approximately 30 minutes of an attack supports a temporal association between hyperkalemia and weakness, it was the only elevated measurement, was obtained during a home blood draw, and was not accompanied by an electrocardiogram or serial potassium measurements. Thus, the available data cannot establish whether hyperkalemia preceded, coincided with, or followed the onset of weakness. Pseudohyperkalemia was also not definitively excluded, which is particularly relevant in the setting of CLL and leukocytosis because leukocyte fragility can produce spurious potassium elevations. Although the remainder of the chemistry profile was similar to baseline and there was no evidence of tumor lysis syndrome or another major metabolic abnormality, paired serum, plasma, or whole-blood potassium measurements and documented specimen-handling conditions were unavailable. CLL may additionally be associated with autoimmune or paraneoplastic neurologic syndromes capable of producing weakness; however, the patient's recurrent stereotyped attacks, lack of progressive neurologic deficits, negative autoimmune evaluation, absence of tumor lysis syndrome, and sustained response to acetazolamide favored a primary disorder of skeletal muscle membrane excitability.

Electrophysiologic evaluation likewise did not provide diagnostic confirmation. Routine nerve conduction studies and needle electromyography showed no active denervation, myopathic changes, or electrical myotonia, but these studies were performed between attacks without specialized exercise testing. No short- or long-exercise testing, provocative exercise protocol, or electrophysiologic testing during an attack was performed because the episodes were sporadic.

Despite these limitations, the patient demonstrated substantial clinical improvement following dietary modification and treatment with the carbonic anhydrase inhibitor acetazolamide [4]. A previously published case of an older adult with hyperkalemic periodic paralysis similarly described stress-induced attacks of variable frequency that initially mimicked transient ischemic attacks because of prominent brainstem muscle weakness, with decreased attack frequency following acetazolamide treatment [10]. Although such reports support the possibility of late-onset presentations, they do not overcome the diagnostic limitations in this case. This case therefore emphasizes the importance of considering hyperkalemic periodic paralysis in older adults with recurrent stereotyped episodes of weakness and obtaining potassium measurements during symptomatic episodes, while recognizing that a single elevated potassium value does not establish causality. In patients with leukocytosis, pseudohyperkalemia should be specifically considered, and specialized electrophysiologic testing and genetic evaluation may provide additional diagnostic evidence when available. Diagnosis should ultimately integrate the clinical phenotype, laboratory findings obtained during attacks, electrophysiologic evaluation, exclusion of alternative diagnoses, and therapeutic response rather than relying on any single diagnostic finding.

## Conclusions

This case illustrates a rare presentation of late-onset episodic generalized weakness associated with a single documented episode of hyperkalemia that was clinically suggestive of hyperkalemic periodic paralysis. However, the diagnosis remains unconfirmed. The patient's exceptionally late age at onset, absence of childhood or adolescent episodes and family history, negative genetic testing, and lack of specialized exercise testing or electrodiagnostic testing during an attack prevent definitive attribution of his episodes to an inherited skeletal-muscle channelopathy. The most appropriate characterization or possible hyperkalemic periodic paralysis rather than confirmed hyperkalemic periodic paralysis is therefore suspected. The documented potassium elevation of 6.5 mmol/L obtained within 30 minutes of an attack and the stereotyped episodic weakness provide supportive evidence, but a single potassium measurement does not establish causality. Similarly, improvement with acetazolamide and dietary modification should be considered supportive and not used as diagnostic confirmation.

In patients with a similar presentation, evaluation should include careful characterization of the weakness phenotype, a complete neurologic examination during and between attacks when feasible, consideration of neuromuscular junction and other neuromuscular disorders, and appropriate metabolic evaluation. In patients with chronic lymphocytic leukocytosis (CLL) or marked leukocytosis, pseudohyperkalemia should be specifically assessed with appropriately handled and paired potassium measurements. If an attack can be captured in the future, specialized exercise testing or electrophysiologic evaluation may provide additional diagnostic evidence.
